# Supplementary material for: Protective Abilities of an Inhaled DPI Formulation Based on Sodium Hyaluronate against Environmental Hazards Targeting the Upper Respiratory Tract
Source: Pharmaceutics. 2022 Jun 22;14(7):1323. doi: 10.3390/pharmaceutics14071323 (PMC9318658; doi:10.3390/pharmaceutics14071323)
Supplement: Supplementary file 1 [file pharmaceutics-14-01323-s001.zip › pharmaceutics-1712713-supplementary.pdf]

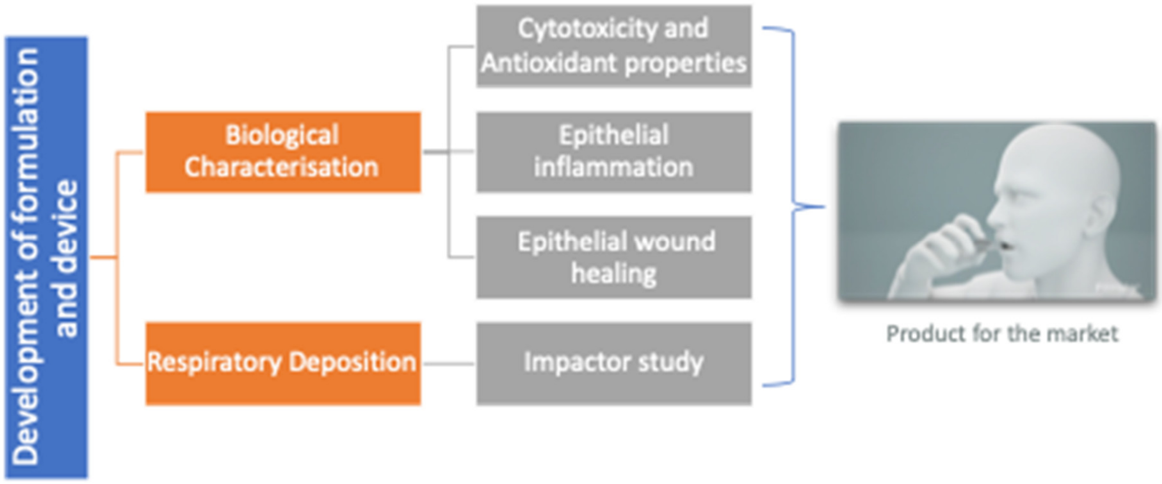

Figure S1. Schematic representation of the scope of the research.

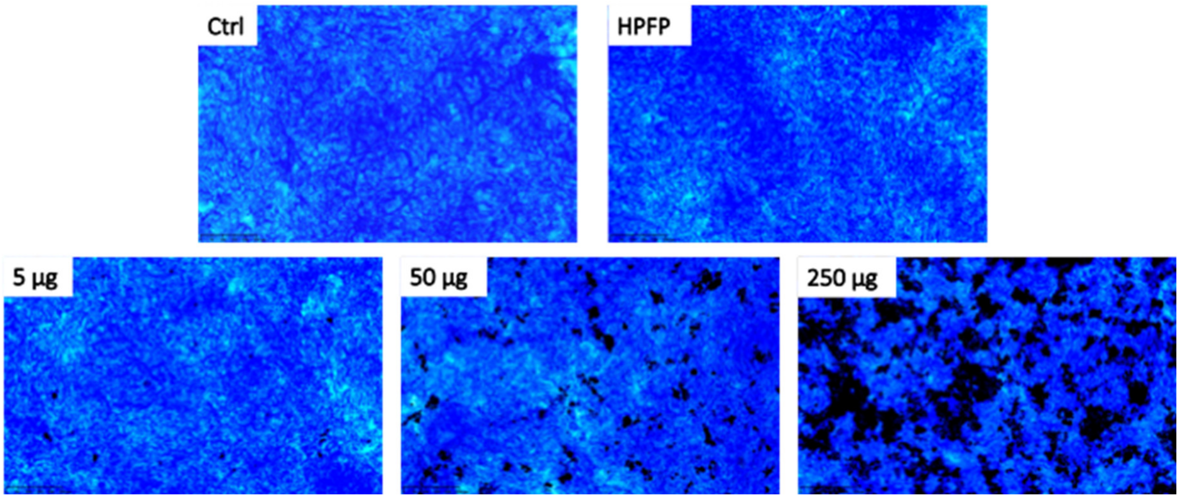

Figure S2. Images of Alcian blue mucus staining of Calu-3 cells grown in ALI model ± urban dust (amounts annotated in the figure).

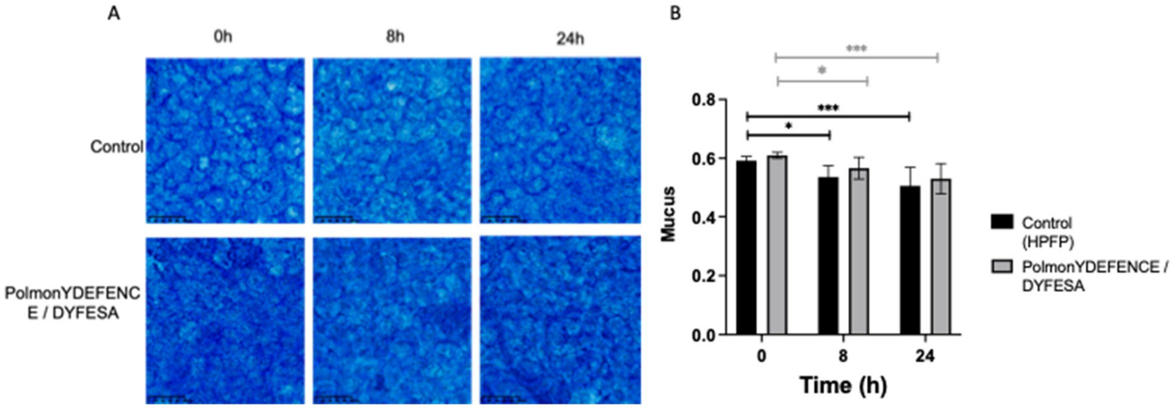

Figure S3. (**A**) Images of Alcian blue mucus staining of Calu-3 cells grown in ALI model ± PolmonYDEFENCE/DYFESA (69 µg). (**B**) Quantification of mucus by image J analysis. Statistical significance was calculated using one-way ANOVA with Dunnett's multiple comparisons tests (* $p < 0.033$; *** $p < 0.0002$).

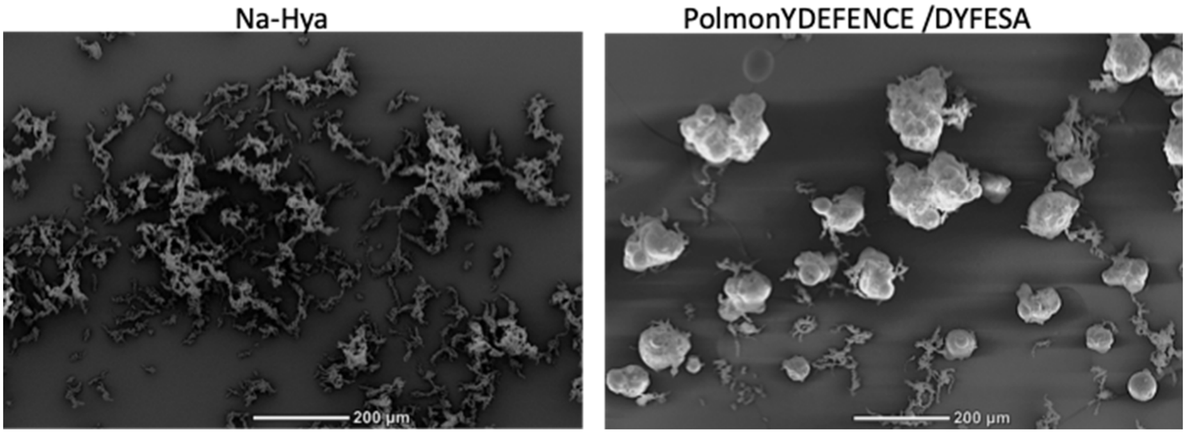

Figure S4. SEM Images of PolmonYDEFENCE/DYFESA and Sodium Hyaluronate (Na-Hya) at 100× magnification.
